# Multiomics: unraveling the panoramic landscapes of SARS-CoV-2 infection

**DOI:** 10.1038/s41423-021-00754-0

**Published:** 2021-09-01

**Authors:** Xin Wang, Gang Xu, Xiaoju Liu, Yang Liu, Shuye Zhang, Zheng Zhang

**Affiliations:** 1 Institute for Hepatology, National Clinical Research Center for Infectious Disease, Shenzhen Third People’s Hospital, The Second Affiliated Hospital, School of Medicine, Southern University of Science and Technology, Shenzhen, Guangdong Province China; 2 Shanghai Public Health Clinical Center, Fudan University, Shanghai, China; 3 Shenzhen Research Center for Communicable Disease Diagnosis and Treatment of Chinese Academy of Medical Science, Shenzhen, Guangdong Province China; 4 Guangdong Key Laboratory for Anti-infection Drug Quality Evaluation, Shenzhen, Guangdong Province China; 5 Shenzhen Bay Laboratory, Shenzhen, Guangdong Province China

**Keywords:** SARS-CoV-2, COVID-19, Multi-omics, Virology, Immune Response, Pathogenesis, Viral infection, Mechanisms of disease

## Abstract

In response to emerging infectious diseases, such as the recent pandemic of coronavirus disease 2019 (COVID-19) caused by severe acute respiratory syndrome coronavirus 2 (SARS-CoV-2), it is critical to quickly identify and understand responsible pathogens, risk factors, host immune responses, and pathogenic mechanisms at both the molecular and cellular levels. The recent development of multiomic technologies, including genomics, proteomics, metabolomics, and single-cell transcriptomics, has enabled a fast and panoramic grasp of the pathogen and the disease. Here, we systematically reviewed the major advances in the virology, immunology, and pathogenic mechanisms of SARS-CoV-2 infection that have been achieved via multiomic technologies. Based on well-established cohorts, omics-based methods can greatly enhance the mechanistic understanding of diseases, contributing to the development of new diagnostics, drugs, and vaccines for emerging infectious diseases, such as COVID-19.

## Introduction

The constant emergence of infectious diseases caused by novel pathogens poses serious threats to public health. During the past two decades, the world has been afflicted with several infectious diseases, including severe acute respiratory syndrome (SARS), Middle East respiratory syndrome, seasonal and avian influenza, Zika virus disease, Ebola virus disease, and the ongoing coronavirus disease 2019 (COVID-19) pandemic [1, 2] caused by SARS coronavirus 2 (SARS-CoV-2) infection [3, 4]. Rapid identification and investigation of the causative pathogens, risk factors for disease outcomes, host immune responses, and pathogenic mechanisms at both the molecular and cellular levels are of great urgency after the emergence of infectious diseases.

**Fig. 1:**
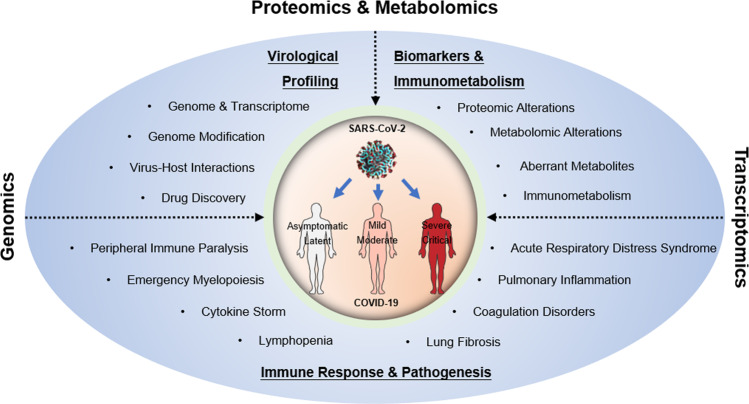
Multiomic technologies facilitate the determination of the virological and immunological characteristics of SARS-CoV-2 infection, the discovery of biomarkers, and the elucidation of COVID-19 pathogenesis. With the use of genomic and transcriptomic-based sequencing, virological characteristics, including the genome, transcriptome, and virus-host interactions of SARS-CoV-2, have been elucidated. Moreover, the characteristics of the immune responses and the pathogenesis of COVID-19, especially in association with severe disease, have been extensively characterized. Systemic and tissue-specific immune disorders, such as lymphopenia, cytokine storm, emergency myelopoiesis, peripheral immune paralysis, and lung inflammation, are strongly associated with the manifestations of severe/critical COVID-19, including acute respiratory distress syndrome, coagulation disorders, and lung fibrosis.

Due to their systemic metrics, multiomic technologies are well placed to enable rapid and unbiased collection of big data and to provide panoramic views and insights into pathogenesis and new strategies to treat diseases [5]. In particular, multiomic technologies have been used to effectively understand the virologic profile and pathogenesis of SARS-CoV-2 infection. The complete genomic sequences of SARS-CoV-2 were obtained directly from clinical samples using high-throughput metagenomic sequencing, contributing to the successful identification of this new pathogen and the analysis of its phylogenetic and evolutionary characteristics [3, 6, 7]. By integrating next-generation/third-generation sequencing, proteomics, metabolomics, and other omics technologies, the viral transcriptome, virus-host interactions, perturbed metabolomics, and biomarkers predicting disease severity have been examined extensively. Single-cell RNA sequencing (scRNA-seq) and immune repertoire (T cell receptor, TCR, or B cell receptor, BCR) sequencing have revealed immune responses from tissue and peripheral blood, which have demonstrated important immunopathological mechanisms of COVID-19. Here, with a systematic review of recent advances enabled by multiomic technologies in virology, immunology, biomarkers, and pathogenic mechanisms of SARS-CoV-2 infection (Fig. 1), we demonstrated that omics-based methods can greatly improve the understanding of diseases and promote the development of new diagnostics, medicines, and vaccines targeting emerging infectious pathogens, including SARS-CoV-2.

## Genome/transcriptome of SARS-CoV-2 and viral–host interactions

SARS-CoV-2 was initially reported to be the pathogen that causes COVID-19 by metagenomic RNA sequencing of bronchoalveolar lavage fluid (BALF) from a patient with pneumonia [7]. Phylogenetic analysis of the viral genome has shown that this new virus belongs to the SARS-like coronavirus family, sharing 89.1% nucleotide similarity with bat-derived strains [7].

### Investigation of the viral transcriptome and genome

High-throughput sequencing technology has been widely adopted in virological research. In addition to second-generation Illumina sequencing, third-generation nanopore-based sequencing can directly generate longer reads of DNA/RNA molecules from clinical specimens without preamplification [8], which makes it possible to quickly detect pathogen nucleic acid sequences [9–11]. By combining Illumina and nanopore sequencing, novel SARS-CoV-2 open reading frames with fusions, deletions, frameshifts, and 41 modified sites have been found [12]. Moreover, viral subgenomes generated by transcription-regulating sequence-dependent template switching have been profiled by mapping hundreds of template switches and dynamic subgenomic landscapes of SARS-CoV-2 [13]. N6-methyladenosine (m6A) is the most abundant type of RNA modification in mammalian cells [14] and viruses [15–17]. Eight m6A modification sites have been identified on the genomic RNA of SARS-CoV-2 through RNA immunoprecipitation sequencing and m6A individual-nucleotide-resolution crosslinking and immunoprecipitation sequencing, indicating the possible regulation of viral infection by m6A methylation modification [18].

The viral RNA structure is also known to be an important factor in viral replication. Recently, an in vivo click selective 2’-hydroxyl acylation and profiling experiment was developed to identify the structural landscape and unreported regulatory regions of SARS-CoV-2 RNA in vivo. Targeting the predicted structural elements with antisense oligonucleotides led to validation of some of their functions during SARS-CoV-2 infection [19]. Therefore, high-throughput sequencing has been used to not only characterize viral genomes quickly and accurately but also reveal the detailed molecular architecture of viral RNA.

### Analysis of virus-host interactions

Viruses can hijack host cells to complete their life cycle, and the host factors required for virus infection may serve as therapeutic targets. For example, SARS-CoV-2 utilizes angiotensin-converting enzyme 2 (ACE2) as its essential receptor [3], and proteases from host cells, including Furin, transmembrane serine protease 2 (TMPRSS2), and cathepsin L1 (CTSL), are required to cleave the spike protein to promote viral entry [20]. Indeed, soluble ACE2 [21], TMPRSS2, and CTSL inhibitors [22, 23] have shown prominent antiviral effects and are potentially applicable in the prevention of SARS-CoV-2 infection and the treatment of COVID-19. Clustered regularly interspaced short palindromic repeats (CRISPR) screening has revealed many important properties of SARS-CoV-2, elucidating new mechanisms of virus-host interactions. Several single guide RNA (sgRNA) libraries (Brunello, GeCKO, and GeCKOv2) have been used to identify host proteins required for SARS-CoV-2 infection in several human or monkey cell lines (Vero-E6, Huh7.5, Huh7.5.1, and A549^ACE2^) [24–27]. TMEM41B has been found to be essential not only for *Coronaviridae* infection but also as a panflavivirus host factor [28]. In addition, several studies have shown that the lysosome protein TMEM106B can be unique to SARS-CoV-2 infection [24, 26, 27]. Another study used chromatin-interacting protein-mass spectrometry to map RNA-binding proteins, performed CRISPR screening of the Vero and Huh7 cell lines, and identified the RNA-binding host factors that affected SARS-CoV-2 infection [29]. However, those screening studies found few common hits; therefore, the identification of viral–host factors requires further research. The cause of these discrepancies may be related to the different cell lines being screened, which cannot mimic natural infection. In the future, primary lung epithelial cells or lung organoids are preferred as screening models to mimic natural SARS-CoV-2 infection.

A variety of cell lines overexpressing ACE2 can support SARS-CoV-2 infection in vitro, but the SARS-CoV-2 tropism in vivo is not very clear. Several scRNA-seq studies of BALF and sputum samples from COVID-19 patients have not detected an abundance of SARS-CoV-2 RNA in airway epithelial cells but have accidentally found viral RNA in immune cells, particularly macrophages and neutrophils, despite the low expression of ACE2 in these immune cells [30–32]. SARS-CoV-2 proteins have also been detected in immune cells in patients’ lungs using immunohistochemistry [31, 33]. It is still unclear how SARS-CoV-2 enters immune cells, whether through passive endocytosis or other receptors such as CD147 [34]. Compared to viral RNA-negative cells, SARS-CoV-2 RNA-positive epithelial cells and neutrophils have been found to have higher levels of interferon-stimulated gene (ISG) expression [30, 31]. In addition to the patient’s lungs, other organs, such as the kidneys, brain, and gastrointestinal (GI) tract, are also significantly affected by SARS-CoV-2 infection [35–37]. In the future, more multiomic studies, such as spatial transcriptome sequencing, imaging mass spectrometry, and NanoString, will be used to further explore the spectrum of viral infection and the response of different cells after infection.

### Identifying potential antiviral targets

An interaction map of viral proteins with host factors can facilitate a better understanding of the virologic mechanisms of SARS-CoV-2 infection, thus benefiting the discovery of antiviral drugs. Affinity-purification mass spectrometry has evolved to become a standard method for detecting protein-protein interactions [38]. Compared with the conventional approach, which uses antibody immunoprecipitation of protein-protein complexes, affinity-purification mass spectrometry is accomplished by tagging epitopes and capturing probes on the bait protein to identify prey proteins without the use of specific antibodies for each new bait protein. Gordon et al. used 26 SARS-CoV-2 proteins [39] labeled with Strep-tags as bait and managed to identify 332 host proteins involved in various protein complexes and biological processes, such as DNA replication, vesicle trafficking, and innate immunity. In addition, they examined the antiviral effects of 69 drugs targeting 62 proteins in vitro and identified two sets of pharmacological agents targeting mRNA translation and sigma-1 and sigma-2 receptors with antiviral activity [39]. Another study used mass spectrometry to reveal the host cell response after SARS-CoV-2 infection, found a substantial increase in translation machinery expression in SARS-CoV-2-infected cells, and showed that two translation inhibitors, cycloheximide and emetine, significantly inhibited SARS-CoV-2 replication [40]. In addition, several groups have applied mass spectrometry to profile proteomic and phosphoproteomic changes following viral infection and then designed and tested candidate drugs based on those omics data [40]. A report showed that infection with SARS-CoV-2 in Vero cells induced activation of casein kinase II and p38 mitogen-activated protein kinase (MAPK) signaling but inhibited mitotic kinases, causing cell cycle arrest [41]. Pharmacological kinase modulators were test, and p38, casein kinase II, cyclin-dependent kinases, AXL, and PIKFYVE kinase inhibitors were identified, which exhibited antiviral activities and represented potential antiviral targets. Another study examined an alveolar type 2 epithelial cell infection model derived from pluripotent stem cells and identified mTOR and MAPK inhibitors as potent antivirals [42]. Together, these studies show that omics technologies can offer swift and sufficient information to identify virus-host interactions and reveal antiviral targets at a systemic level.

## Proteomic/metabolomic features associated with severe COVID-19

SARS-CoV-2 infection evokes highly dynamic and heterogeneous diseases. Accurate diagnosis and prognosis prediction are important for appropriate individualized therapy, which could benefit from biomarker studies. Although risk factors associated with the severity of COVID-19 have been reported previously [43, 44], knowledge of their predictive efficacy is still limited. With the use of mass spectrometry, proteomic, metabolomic and lipidomic changes in patients with COVID-19 have been extensively studied (Table 1), potentially revealing biomarkers and pathogenesis information for classifying disease severity and predicting outcomes.

**Table 1:** Proteomic and metabolomic changes in plasma from severe COVID-19 patients.

Features	Key altered biomarkers	References
Inflammatory responses	↑ APPs (SAA-1, SAA-2, SAA-4, ORM1, ORM2, S100A8/S100A9, SERPINA3, SAP/APCS, CRP, TKT); ↑ LCP1/LPL; ↑CFI	[45, 48–50]
Complement system	↑ C6; ↑CFB; ↑CFP, CPN1; ↑mannose	[45]
Platelet degranulation/coagulation	↓ PPBP, ↓PF4, ↓serotonin, ↓ HRG, ↓GPLD1, CLEC3B; ↓F2; ↓F13A1, F13B; ↓ PROC; ↓SERPINA5; ↑SRGN, VWF; ↑FGA, FGB	[45, 48]
Vessel damage	↑ AGT; ↑FBLN5, NID1; ↑SERPINB1; ↑NRP1; ↓SERPINA4	[48]
Other proteins	Dysregulation of multiple apolipoproteins (APOA1, APOA2, APOH, APOL1, APOD, APOM); ↓ALB; ↓APOA1, APOC1; ↓GSN; ↓TF; ↓FETUB, ↓CETP, PI16; ↑AZGP1; ↑kynurenate, kynurenine	[45, 49]
Amino acid metabolism	↓ >100 amino acids; ↓ arginine metabolism (glutamate, arginine, N-(l-arginino)-succinate, citrulline, ornithine, glutamine, 2-oxoglutarate, N-acetyl-L-glutamate, urea, fumarate); ↓ tryptophan; ↓ valine; ↓ proline; ↓ isoleucine; ↓ carbamoyl phosphate	[4, 45, 47, 72]
Lipid metabolism	↓ lipids, neutral lipids; ↓ sterol, cholesterol; ↓ sphingolipids; ↓ palmitoylcarnitine; ↓ stearoylcarnitine; ↓ oleoylcarnitine; ↓ acylcarnitines; ↓ glycerophospholipids; ↓ choline; ↑ phosphocholine; ↑ 21-hydroxypregnenolone	[4, 49, 59]
Other factors connected to metabolism	↑ HIF-1 signaling pathway; ↑ reactive oxygen species; ↑ lactate dehydrogenase; ↑ succinate; ↓ TCA cycle metabolites; ↓ malic acid; ↓ D-Xylulose 5-phosphate; ↓ guanosine monophosphate; ↓ dihydrouracil; ↓ itaconic acid	[4, 54, 59, 72]

*APP* activated acute phase protein, *SAA* serum amyloid A, *ORM1* orosomucoid-1/alpha-1-acid glycoprotein-1, *SERPINA3* alpha-1-antichymotrypsin, *SAP/APCS* serum amyloid P-component, *CRP* C-reactive protein, *TKT* transketolase, *LCP1/LPL* lymphocyte cytosolic protein 1/L-plastin, *CFI* complement factor I, *C6* complement 6, *CFB* complement factor B, *CFP* properdin, *CPN1* carboxypeptidase N catalytic chain, *PPBP* platelet-expressing chemokines proplatelet basic protein, *PF4* platelet factor 4, *HRG* histidine-rich glycoprotein, *GPLD1* glycosylphosphatidylinositol-specific phospholipase D1, *CLEC3B* C-type lectin domain family 3 member B, *F2* prothrombin, *F13A1 and F13B* thrombin-activation factor, *PROC* protein C, *SERPINA5* serpin family A member 5, *SRGN* serglycin, *VWF* von Willebrand factor, *FGA* fibrinogen alpha, *FGB* fibrinogen beta, *AGT* angiotensinogen, *FBLN5* fibulin-5, *NID1* nidogen 1, *SERPINB1* serpin family B member 1, *NRP1* neuropilin-1, *SERPINA4* serpin family A member 4, *APOA1* apolipoprotein A1, *APOH* apolipoprotein H, *APOL1* apolipoprotein L1, *APOD* apolipoprotein D, *APOM* apolipoprotein M, *APOC1* apolipoprotein C1, *ALB* albumin, *GSN* gelsolin, *TF* transferrin, *FETUB* fetuin-B, *CETP* cholesteryl ester transfer protein, *PI16* peptidase inhibitor 16, *AZGP1* zinc-α2-glycoprotein-1, *HIF1A* hypoxia-inducible factor 1 subunit alpha, *TCA* tricarboxylic acid.

### Proteomic and metabolomic alterations

Several studies have characterized proteins and metabolites differentially expressed in COVID-19 patients [4, 45–49]. Some of the proteins with the most enhanced serum expression in severe COVID-19 cases include acute phase proteins [45, 46, 50], which corresponds to their roles in inflammation, infection, or tissue injury [51]. Recently, a panel of five proteins with downregulated expression, namely, albumin (ALB), apolipoprotein A1 (APOA1), apolipoprotein C1 (APOC1), gelsolin (GSN), and transferrin (TF), was identified as a core signature associated with the severity of COVID-19 [46]. In particular, low levels of plasma APOA1 and GSN have already been reported in sepsis and systemic inflammatory response syndromes [52, 53].

Enrichment analysis of differentially expressed plasma proteins showed dysregulated pathways related to inflammation, immune cell migration and degranulation, the complement system, coagulation cascades, and energy metabolism in severe versus mild COVID-19 cases [49]. A pivotal metabolic shift in COVID-19 patients is represented by a disproportionate reduction in nutrient circulation [47]. In particular, there is a preferential depletion of metabolites associated with the TCA cycle with increasing disease severity [47, 54], which has been confirmed by multiple independent studies [45, 55, 56]. It should be noted that the levels of a number of plasma fatty acid metabolites from recovered COVID-19 patients with impaired lung function were not normalized, indicating a lack of complete recovery [54, 57]. With the use of multiomics in several studies, potential biomarker panels of plasma proteins, metabolites, and lipid molecules have been developed to assist in the clinical diagnosis and prognosis of COVID-19 [4, 48, 49, 58, 59]. For example, Shen et al. discerned severe COVID-19 via the expression signatures of 22 serum proteins and 7 metabolites, with 93.5% accuracy [45]. Shu et al. predicted fatal outcomes of severe COVID-19 with cholesteryl ester transfer protein, S100A9, and C-reactive protein levels [49]. Overall, rapid and reliable proteomic and metabolomic studies can improve the clinical diagnosis and prognosis prediction of severe cases.

Significant dysregulation of the lung proteome has been characterized in SARS-CoV-2 infection [60]. To examine the proteomic landscape of multiple organs, Nie et al. analyzed 144 autopsies of seven organs in COVID-19 deaths [61]. They reported significantly upregulated expression levels of cathepsins such as CTSL rather than of ACE2 in the lung [61], which has also been confirmed by others [62], suggesting the important role played by cathepsins in the increasing severity of COVID-19. In addition, the inflammatory response modulator S100A8/S100A9 has been found to be strongly and widely expressed in the lungs [62] and multiple extrapulmonary organs, accompanied by dysregulated glucose and fatty acid metabolism [61]. Perturbed pathways involved in hypoxia, angiogenesis, blood coagulation, and fibrosis have also been identified in multiple organs of COVID-19 patients [61, 63]. Therefore, systemic proteomic and metabolic dysregulation occur along with multiple organ damage, and these metadata highlight the pathogenesis of COVID-19.

### Immunometabolism

Metabolites not only provide energy and substrates for cell growth and survival [64] but also regulate the function of immune cells [65, 66]. The immune response and metabolism regulation are highly integrated and interdependent [67]. Based on immune-metabolic profiling, the expansion of metabolically distinct T cells and myeloid-derived suppressor cells occurs, particularly during acute COVID-19 [68, 69]. Longitudinal multiomic analyses of peripheral blood samples of hospitalized patients have revealed that severe COVID-19 is characterized by an increase in metabolically hyperactive plasmablasts [70]. Expansion of interferon (IFN)-activated circulating megakaryocytes and erythropoiesis featuring hypoxic signaling has also been identified in critical cases [70]. Metabolic reprogramming of immune cells is critical for inflammatory responses [71]. Targeting metabolism may be a potential therapeutic strategy for modulating proinflammatory cytokine release and immune cell survival, which has been confirmed in COVID-19 [68, 72]. The addition of arginine or epacadostat markedly modulates the release of proinflammatory cytokines by peripheral blood mononuclear cells isolated from SARS-CoV-2-infected rhesus macaques [72]. In addition, T cell apoptosis in severe COVID-19 is inhibited in vitro by targeting voltage-dependent anion channel 1 oligomerization, which is associated with mitochondrial dysfunction and apoptosis [68]. Thus, evidence emerges that targeting specific metabolic events can strengthen antiviral immune responses while limiting nonproductive and adverse inflammation [66].

## Immune landscape of COVID-19

**Fig. 2:**
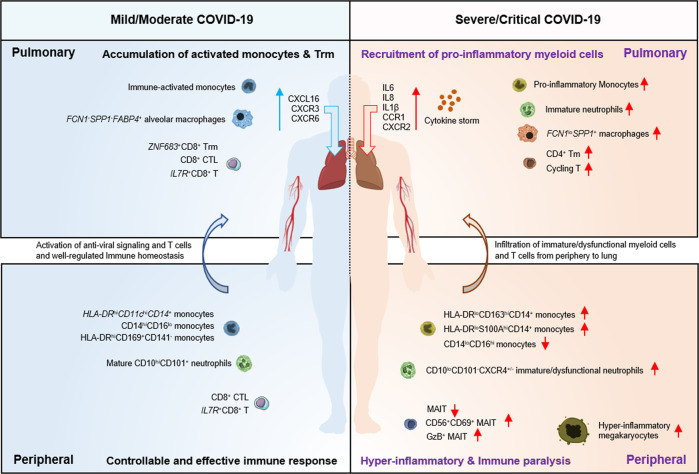
Immune dysfunction of the lung and peripheral compartments in mild and severe COVID-19. Using scRNA-seq and mass cytometry, lung and peripheral immune responses have been examined in patients with mild and severe COVID-19. In the peripheral blood of patients with severe COVID-19, immature/dysfunctional myeloid cells (e.g., HLA-DR^lo^CD163^hi^CD14^+^ monocytes, HLA-DR^lo^S100A^hi^CD14^+^ monocytes, and CD10^lo^CD101^-^CXCR4^+/–^ immature/dysfunctional neutrophils), GzB^+^ MAIT cells, CD56^+^CD69^+^MAIT cells, and hyperinflammatory megakaryocytes accumulated, while nonclassical monocytes (CD14^lo^CD16^hi^) and total MAIT cells are depleted. Recruitment of immature/dysfunctional myeloid cells and peripheral T cells to pulmonary sites further promotes the cytokine storm and the inflammatory environment during severe COVID-19. In contrast, mild cases tend to have well-controlled immune homeostasis, including the appropriate activation of myeloid cells, T cells, and antiviral signaling as well as clonal expansion of resident T cells in lung tissues.

The immune response, especially through the heterogeneous respiratory immune microenvironment, is associated with the severity of COVID-19 and clinical outcomes of SARS-CoV-2 infection. The application of multiomic approaches, including scRNA-seq and high-dimensional flow cytometry, has facilitated the exploration of heterogeneous immune landscapes in COVID-19 patients (Fig. 2).

### Immune responses in the upper respiratory tract

The upper respiratory tract is the initial site of SARS-CoV-2 infection [73–76] and is the first barrier against invasive pathogens. The virus is constrained in most asymptomatic and mild COVID-19 patients, and severe pneumonia occurs as the situation deteriorates when the virus reaches the lower respiratory tract after the infection spreads beyond the nasopharyngeal site, highlighting the significance of a holistic understanding of the virus dynamics and host immune response at the nasopharyngeal site. Several scRNA-seq studies have examined nasopharyngeal samples from COVID-19 patients or organoids infected with SARS-CoV-2, including human bronchial epithelial cells at the air–liquid interface [77] and human-derived tracheo/bronchial epithelial cells that form the mucociliary epithelium [78]. SARS-CoV-2 primarily targets ciliated cells in the upper respiratory tract [77] and depletes mature epithelial cells but expands proliferation of secretory cells with diversified phenotypes [79]. Notably, diminished epithelial antiviral immunity, with delayed IFN production and weakened IFN-induced responses, has been noticed in the nasal mucosa of severely ill COVID-19 patients, suggesting that impaired local IFN responses are involved in uncontrolled viral infection [78, 79]. However, we recently found that the immune responses in the upper respiratory tract were significantly strengthened in asymptomatic carriers infected with SARS-CoV-2, likely helping eliminate the virus, ultimately stopping the virus from infecting the lungs and causing disease. In addition, there is enhanced crosstalk between epithelial cells and myeloid cells and an increase in T lymphocyte levels and clonal expansion, suggesting that a coordinated early immune response in situ is effective in eliminating SARS-CoV-2 and preventing disease (Xu, 2021, unpublished data). Future assays should address the local dynamics of IFN responses during SARS-CoV-2 infection.

### Immune responses in the lower respiratory tract and lung

To some extent, SARS-CoV-2 infection of the lower respiratory tract contributes to severe clinical outcomes, including acute lung injury, acute respiratory distress syndrome and even death. Therefore, a comprehensive analysis should be conducted on the lung microenvironment in patients with COVID-19 and various illnesses. Two reports characterized the BALF immune signatures from COVID-19 patients using scRNA-seq or RNA-seq during the initial stages of the COVID-19 pandemic and revealed the disruption of bronchoalveolar epithelial barriers, extensive immune infiltration, hypercytokinemia, and an increase in IFN-induced responses [32, 80]. Remarkably, lung monocytes/macrophages from severely/critically ill patients were found to have high expression of *FCN1* and *SPP1*, whereas expression of the alveolar macrophage marker *FABP4* was identified in monocytes/macrophages from mildly/moderately ill patients [32]. Further analysis found that patients with severe/critical COVID-19 expressed higher levels of inflammatory cytokines, including interleukin (IL)-1β, IL-6, and IL-8, in the lungs [32]. More importantly, extremely high levels of chemokines, including C-C motif chemokine ligand (*CCL*) *2*, *CCL3*, *CCL4*, and *CCL7*, have been found to be expressed in patients’ lung monocytes/macrophages [32, 81]. Other studies have also shown high expression of C-C chemokine receptor (*CCR*) *1*, *CCR2*, and *CCR5* (CCL2/CCL3 receptors) in lung macrophages and neutrophils, especially in critically ill patients [82, 83]. In addition, enhanced epithelium-immune cell interactions have been observed in critically ill COVID-19 patients, especially among highly activated monocytes, monocyte-derived macrophages and cytotoxic T lymphocytes [82]. Consistently, the upregulation of mucin 5AC (*MUC5AC*), a major component of secreted mucins, has also been found in club cells from COVID-19 patients [84]. Collectively, these results indicate increased recruitment of inflammatory monocytes and neutrophils into the lungs of patients with severe COVID-19, which subsequently leads to immune-mediated epithelial damage and bronchoalveolar epithelial dysfunction.

Unlike the hyperactivation of pulmonary monocyte-macrophages, immune responses of T cells are dysfunctional in patients with severe COVID-19. Examination of the BALF from COVID-19 patients has revealed fewer CD8^+^T cells but more proliferating T cells in severe/critical cases [32, 85]. In patients with mild COVID-19, tissue-resident memory (T_RM_) CD8^+^ and CD4^+^ T helper-17 (Th17) cells are characterized by increasing (presumably antigen-driven) clonal expansion and strengthened effector functions, whereas their counterparts remain more naive/intermediate in critical COVID-19 [86]. Another study integrated molecular, functional, and clinical data from lung and peripheral blood memory CD8^+^ T cells and found that correlations may exist between immune silencing and severe clinical manifestations/fatal outcomes [87]. These data addressed the differential roles of lung-infiltrating T cells in mild versus severe COVID-19 and elucidated that tissue-resident T cell responses are crucial for controlling viral infection and preventing diseases.

In addition to perturbed lower respiratory tract and lung immune landscapes for COVID-19 patients, persistently increased levels of IFN-α/β and IFN-λ in the lungs of patients with severe COVID-19 can be another key issue, given their potential to interfere with lung epithelial cell repair and recovery, especially IFN-λ [88–90]. In contrast, higher local IFN responses identified in mild COVID-19 may help constrain viruses in the airways [79, 86] at an early stage of infection. Therefore, the differences in magnitude and duration of IFN production may lead to a significantly different outcome from SARS-CoV-2 infection.

### Immune pathogenesis

Omics studies have revealed immunopathological mechanisms underlying severe COVID-19, which may be greatly significant. Transcriptomic and histological studies have shown heterogeneous immunopathological profiles in lung autopsies of deceased COVID-19 patients [91]. One pattern showed limited lung damage despite higher levels of ISGs, cytokines and viral loads, particularly in patients who died earlier. The other pattern showed lower levels of ISGs and viral loads but abundant immune infiltration along with massive pulmonary lesions in patients who died later [91]. Other studies have found persistent enrichment of T cells and myeloid cells in the alveolar space in most patients with severe COVID-19 [92]. Both bulk RNA-seq and scRNA-seq profiling have suggested that SARS-CoV-2 infection may induce alveolar macrophages to produce T cell chemoattractants. As a feedback loop, recruited T cells produce IFN-γ, which induces macrophages to release more inflammatory cytokines and further promotes the activation of T cells [92], thus creating a circuit between active macrophages and T cells and amplifying SARS-CoV-2-induced pneumonia.

Another scRNA-seq study tracked T cell clones across tissues and conducted interactome analyses [93]. The authors demonstrated that clonally expanded and granulocyte-macrophage colony-stimulating factor (GM-CSF)-producing tissue-resident memory-like T_H_17 cells were highly associated with a severe clinical course resulting from crosstalk with lung macrophages and cytotoxic CD8^+^ T cells [93, 94]. In addition, the complement system has recently been shown to be highly active in lung epithelial cells infected with SARS-CoV-2 and associated with the severity of the disease [95]. These findings suggest that the involvement of a positive feedback loop among lung monocytes/macrophages and T cells, GM-CSF produced by expanded Trm17 cells, local complement activation, endothelial disruption, and vascular diseases may be specific in driving COVID-19 pathogenesis.

### Single-cell landscape of peripheral immune cells

Early in the COVID-19 pandemic, perturbation of peripheral blood cells was observed in patients with severe COVID-19, including lymphopenia [96, 97], thrombocytopenia [98], and coagulation disorders [99–101]. Later, several omic studies reported an overall immune-paralyzed compartment in the peripheral blood compared with the aberrant immune hyperactivation in the lungs of patients with severe COVID-19 [85, 102, 103]. These studies revealed the dynamic perturbations of peripheral immune cells as the severity of COVID-19 (Table 2) increased, including expanded monocytes and cycling T cells and depleted natural killer cells, T cells, mDCs, and pDCs [85, 102], which was subsequently confirmed by other conventional characterization strategies.

**Table 2:** Dynamic changes in peripheral immune cells and transcriptional features over the course of COVID-19.

Immune cell types and features	Early stage		Peak stage of the disease		References
	Mild	Severe	Mild	Severe	
*CD4/CD8 T cells*					
CD4 naive	ns	ns	ns	ns/–	[85, 117, 136, 153]
CD4 memory	ns	ns	ns	ns/–	
CD4 T_fh_	ns	ns	ns	+	
CD4 T_reg_	ns	+	ns	+	
CD8 naive	ns	–	ns	––	
CD8 GZMK^+^ memory	ns/+	ns	+	+	
CD8 GZMB^+^ CTL	–		–	++	
CD8 proliferation	+	+	+	++	
Functional TCR expansion	Yes	None	+/++	+	
*Innate T cells and NK cells*					
MAIT cells	ns	ns/–	ns/–	––	[85, 117, 122, 153–155]
γδ T cells	ns	ns/–	ns/–	––	
NKT cells	ns	ns/–	ns/–	––	
NK cells	ns	ns/–	ns/–	ns/–	
*B cells and features*					
B naive	ns	ns	ns	ns	[70, 110, 117, 124, 135, 153]
B memory	ns	ns	ns	––	
Plasmablasts	+	+	+	++	
IgG1-BCRs with low SHM	Yes	None	+	+	
*Dendritic cells (DCs)*					
pDC	ns	–	ns/–	–––	[102, 110, 125, 156–159]
mDC1/mDC2	ns	–	ns/–	–––	
*Myeloid cells and features*					
CD14^+^ monocytes	ns	ns	ns	++	[31, 85, 104, 105, 110, 117]
Intermediate monocytes	+	+	+	+	
CD16^+^ monocytes	–	ns/–	–	––	
Immature neutrophils	ns	ns	ns	+++	
MDSC-like features	None	+	ns/+	++	
Antigen presentation	ns	Poor	ns	Poorer	
IFN response	+	+++	++	+	
Cytokine production	ns/–	Poor	Poor	Poorer	

*T*_*fh*_ T follicular helper cell, *T*_*reg*_ regulatory T cell, *NK* natural killer, *MAIT* mucosal-associated invariant T, *pDC* plasmacytoid dendritic cell, *mDC* myeloid dendritic cell, *ns* not significant.

+/++/+++ indicates the degree of increase; −/−−/−−− indicates the degree of decrease; Yes/None indicates the existence of specific features.

One prominent feature is the remodeling of the myeloid cell compartment, as indicated by the accumulation of immature and dysfunctional monocytes/neutrophils in patients with severe COVID-19, revealed by scRNA-seq and mass cytometry [104, 105]. These peculiar monocyte clusters with *HLA-DR*^lo^*S100A*^hi^ and *HLA-DR*^lo^CD163^hi^ phenotypes have been found to have a strong association with the severity of COVID-19 [105]. Severe COVID-19 is also characterized by emergency myelopoiesis, manifested by an increase in the levels of CD10^lo^CD101^-^CXCR4^+/−^ immature neutrophils, low-density neutrophils, *FUT4*^+^*CD63*^+^*CD66b*^+^ proneutrophils and *ITGAM*^+^*CD101*^+^ preneutrophils [105]. These mature *CD274*^+^*ZC3H12A*^+^ neutrophils are reminiscent of granulocytic myeloid-derived suppressor cell-like cells, which can perform immunosuppressive or antiinflammatory functions. In addition, massive release of S100A8/S100A9 calprotectin (a ligand of TLR4 and RAGE [106]) has been observed in plasma from patients with severe [107] rather than mild COVID-19 [85, 104, 105]. As calprotectin can regulate the production of TNF-α [106] and CXCL8 [108] and promote NF-κB activation [109], elevated levels of the S100A8/S100A9 heterodimer may trigger a harmful hyperinflammatory loop in patients with severe COVID-19.

With the exception of the significantly altered myeloid compartment in COVID-19 patients, the links between IFN-induced responses and the severity of COVID-19 are less understood, thus arousing controversy. In peripheral blood, type I IFN deficiency is considered a hallmark of severe COVID-19, characterized by the lack of IFN-β production and low level of IFN-α production and activity [85, 110]. Insufficient IFN-induced responses observed among patients with severe COVID-19 compared with those among patients with mild/moderate COVID-19 also support this conclusion [85, 110]. Accordingly, we and others have also demonstrated suppressed immune signaling and cytokine production modules in peripheral myeloid cells of patients with severe COVID-19 [85], which is proof of overall impaired immune function. One study has shown that the compromised type I IFN response may be caused by an inborn error of type I IFN immunity in patients with severe disease [111]. However, Povysil et al. recently tested 13 predicted loss-of-function (pLOF) variants [111] in a larger cohort consisting of 1934 COVID-19 cases (713 with severe disease and 1221 with mild disease) and found only one rare pLOF mutation in these genes in 713 severe COVID-19 cases and no pLOF variant enrichment in severe cases compared to that in controls or mild COVID-19 cases [112]. Intriguingly, IFN-reactive antibodies or serum antibodies that bind to Fc receptors have been shown to antagonize the IFN response in patients with severe COVID-19 [113, 114]. However, Meisel et al. [115] recently described four patients with autoimmune-polyendocrine-syndrome type 1, and preexisting high levels of neutralizing antibodies against IFN-α and IFN-ω were associated with only mild symptoms of COVID-19. Moreover, longitudinal analyses comparing severe and moderate cases have indicated that the level of type I IFN in plasma from severe COVID-19 cases was lower in the first few days following symptom onset but stayed higher later, compared with a decline in moderate cases [116, 117].

Another prominent feature of severe COVID-19 is lymphopenia [85, 96]. Although lymphopenia is a characteristic shared by various respiratory viral infections, such as influenza and respiratory syncytial virus infections, it is transient and usually lasts for 2–4 days [118, 119]. In contrast, COVID-19-associated lymphopenia can persist much longer and is more selective for T cell lineages. Using scTCR tracking analysis, we and others have proposed that lymphopenia could be caused by the recruitment of peripheral T cells into the lungs of severely ill patients [85]. This view can be supported by the restored numerical reduction in peripheral immune cell subsets following the resolution of serious disease. Others speculate that high levels of cytokines (such as IL-6, IL-10, or TNF) [120, 121] and proapoptotic molecules might contribute to the depletion of T cells. Indeed, large-scale scRNA-seq has revealed that SARS-CoV-2 RNAs could be detected in multiple epithelial [30, 31] and immune cells [31], indicating the possibility of viral infection-triggered cell death resulting in lymphopenia and immune paralysis in severe COVID-19.

Omics studies have also found loss of innate-like T cells and innate lymphoid cells as part of lymphopenia associated with severe COVID-19 [122]. One study enrolled 208 patients with COVID-19 [122] and reported a significant reduction in mucosa-associated invariant T (MAIT) cells in the blood and its strong association with the severity of the disease. Another study also confirmed the loss of innate-like T cell subsets, including MAIT cells, γδ T cells, and NKT cells [86, 123].

Furthermore, omic studies have revealed the protective immune response associated with the control of SARS-CoV-2 infection. Analysis of peripheral leukocytes by mass/flow cytometry [124, 125] and scRNA-seq [126] has shown a significant increase in the proportion of plasmablasts and CD8 effector T cells in all COVID-19 patients. It should be noted that the kinetics of the CD8 effector T cell response could be prolonged and continued for up to 40 days after the onset of symptoms [125]. During recovery from COVID-19, patients generally exhibit robust T cell activation and differentiation signatures at the whole-transcriptome level [126]. Moreover, CD8^+^ T cells kill virus-infected cells through TCR-mediated recognition of viral antigens and create a unique TCR repertoire for each patient. One study revealed that the most expanded clone of recovered subjects was TRAV8-6-TRAJ45:TRAV7-8-TRBJ2-1 [103]. COVID-19 patients with severe hyperinflammatory disease typically exhibit a skewed TCR profile corresponding to superantigen activation [127–129], and the clone with the highest frequency is TRAV12-2-J27-TRBV7-9-J2-3 [130].

Increased numbers of plasmablasts/plasma cells and activated B cells and decreased numbers of resting memory B cells have been observed through scRNA-seq characterization of the B cell compartment in patients with acute COVID-19 [85, 131]. Other studies using high-dimensional flow cytometric profiling have shown activation of prominent extrafollicular B cells and autoimmune-associated BCR features in critically ill patients [132, 133]. Interestingly, the antibody response against the SARS-CoV-2 spike protein receptor-binding domain (RBD) is predominantly mediated by near-germline antibodies with low levels of somatic hypermutations (SHMs) [134]. Moreover, BCR repertoire sequencing has shown that the early recruitment of B cells with a low-SHM signature is correlated with seroconversion of anti-SARS-CoV-2 IgG [135]. Recently, we and others have also found evidence that early engagement of the humoral antiviral response may be crucial to curing the infection and preventing severe disease [136, 137] (Xu, 2021, unpublished data).

### Abnormal coagulation

Abnormal blood coagulation is another major peripheral manifestation of COVID-19, and the frequency of thrombocytopenia is correlated with the severity of COVID-19. In a subset of patients with COVID-19, the host inflammatory response is uncontrolled and causes systemic inflammation [138], with an elevated proportion of dying patients exhibiting progressive thrombocytopenia. Along with the decrease in platelet levels, researchers have also found increased blood clotting in small vessels of multiple tissues in COVID-19 patients, including those of the lungs, heart, and liver. It is unlikely that direct infection of megakaryocytes, as seen in dengue, is responsible for this alteration. Increased levels of circulating precursors, including megakaryocyte-erythrocyte progenitors, may not only represent a secondary response to the increased consumption of platelets but also reflect emergency megakaryopoiesis caused by inflammation [139]. The expansion of IFN-activated circulating megakaryocytes has also been identified in severe COVID-19 by bulk and single-cell RNA-seq [70]. Furthermore, a distinct gene expression profile of circulating platelets has been reported in COVID-19 patients [140] and was particularly associated with protein ubiquitination, antigen presentation, and the mitochondrial dysfunction pathway. These changes might be caused by the direct activation of platelets by SARS-CoV-2 [140] or by the peripheral inflammatory environment [140].

### Injury of other organs

In addition to characterizing respiratory, pulmonary, and peripheral pathogenesis, a few studies have examined other organs, including GI, kidney, liver, bone marrow (BM), and heart tissues, to reveal the potential systemic damage caused by COVID-19 [141]. Interestingly, a proinflammatory response in the GI tract is largely absent despite the detection of SARS-CoV-2, and there is a significant reduction in disease severity and mortality in patients exhibiting GI symptoms [142]. Furthermore, the temporal transcriptional activity of SARS-CoV-2 has been found to be closely associated with disturbance of the gut microbiome in COVID-19 patients [143], and oral and fecal microbial diversity have been shown to decrease significantly in COVID-19 patients compared with those in healthy controls [144].

Importantly, previous studies have shown that COVID-19 can significantly affect the hematological and immunological systems, resulting in lymphopenia, thrombocytopenia, immature/dysfunctional neutrophil accumulation, etc., suggesting dysregulated hematopoiesis in the BM of COVID-19 patients. Using RBD subdomain 1 of the spike protein of SARS-CoV-2 (RBD-SD1) as a probe to investigate the potential tropism of SARS-CoV-2 in 33 types of normal human tissue, RBD-SD1 has been found to strongly interact with BM cells [145]. We recently conducted a scRNA-seq analysis (Wang et al., Cell Discovery, accepted) to characterize the BM mononuclear cell atlas in COVID-19 patients. The results showed that hematopoietic stem cells and multipotent progenitor cells are immune activated, nonproliferating, and apoptotic in patients with COVID-19. More importantly, levels of lymphoid-committed progenitors and pre-B/pro-B cells are dramatically reduced in patients with severe COVID-19 but not in mild cases. In addition, immature/dysfunctional-like granulocyte-monocyte progenitors have been found to accumulate in patients with severe COVID-19. In summary, these results indicate that patients with severe COVID-19 are characterized by dysregulated hematopoiesis.

Recently, the pathological features of COVID-19 have been studied using single-nucleus RNA-seq of lung tissues and scRNA-seq data from kidney, liver, and heart autopsies of COVID-19 patients [146, 147]. The results have shown that few viral RNA reads are present in the kidneys, liver, or heart, while another study reported viral reads in extrapulmonary tissues of COVID-19 patients [148]. Notably, a significant reduction in the proportion of cardiomyocytes and pericytes and an increase in the levels of vascular endothelial cells have been found in patients with COVID-19 [147]. However, these studies were generally preliminary, and future studies should be performed to monitor and evaluate affected nonrespiratory organs in patients who are suffering or even recovering from COVID-19.

## Conclusions and perspectives

**Fig. 3:**
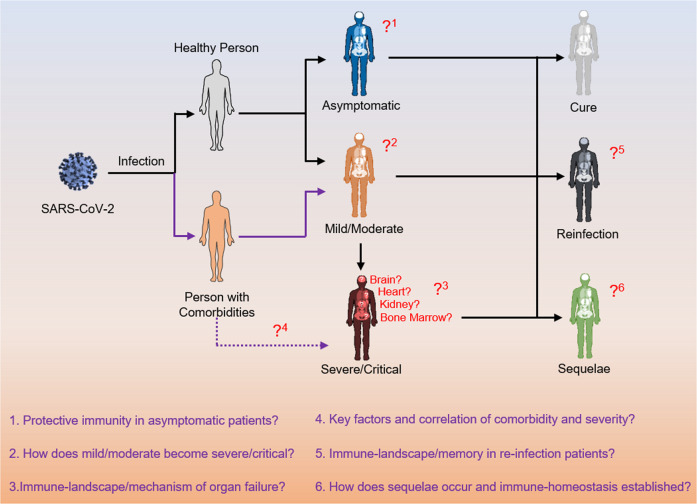
Remaining questions regarding immunity related to COVID-19. There are many unresolved immunological questions regarding the pathogenesis and complications related to COVID-19. Of note, little is understood about protective immunity in asymptomatic patients, key early factors associated with disease severity, risk factors that affect COVID-19 outcomes, immune responses associated with SARS-CoV-2 reinfection, and long-term immune memory in convalescent COVID-19.

The value and advantages of omics technologies are manifested, as they are extensively used by researchers to obtain a fast, multidimensional, and comprehensive understanding of the etiology, immunology, and pathology of SARS-CoV-2/COVID-19. The results obtained with multiomic and traditional methods can complement each other, thus ensuring the reliability of the omics findings. Although omics studies have contributed to a comprehensive understanding of SARS-CoV-2 infection, several important gaps remain to be addressed in future studies (Fig. 3). First, virological and immunological events in the early stages of infection and the long-term stages of recovery should be further analyzed because much of the current understanding of COVID-19 comes from the onset of the disease and short-term follow-up studies of recovered patients. Second, COVID-19 is a systemic disease involving multiple-organ infection and pathological changes. To date, most studies have focused on respiratory, pulmonary, and peripheral changes associated with COVID-19, but little is known about how the infection affects the BM, intestines, brain, heart, kidneys, and other organs. Finally, much remains unknown regarding how age, sex, preexisting diseases, and other factors affect the progression of COVID-19, the reasons why SARS-CoV-2 induces special syndromes in certain populations, and the unique characteristics of COVID-19 compared to other viral infections.

In the future, advances in omics methodologies, such as scATAC-seq, spatial transcriptomics, glycomics, multiomics integration technology, BCR/TCR databases, and antigen-specific prediction algorithms, will certainly offer new opportunities to address unresolved issues in unprecedented detail. For example, spatial transcriptomics [149–151] and genomics [152] have revealed the high-resolution immune environment in patients with COVID-19, which is determined by spatial signaling and cell–cell interactions. Spatial transcriptomics/genomics and organoid/assembloid infection models can help us understand how the immune response is initiated and then dysregulated. Furthermore, as they are increasingly adopted [47, 70, 153], multiomics and integrated analytics will certainly play a more significant role to comprehensively elucidate pathogen activities, host immune responses, and pathogenic mechanisms from multiple perspectives.

In summary, multiomics has significantly improved our understanding of SARS-CoV-2 infection, including the transcriptome/epitranscriptome/proteome of the virus, virus–host interactions, the immune landscape, and proteomic/metabolic biomarkers. It is expected that future studies based on well-established longitudinal cohorts and additional omics technologies will provide a comprehensive and multidimensional view of SARS-CoV-2 and COVID-19, which would greatly facilitate the design and optimization of prophylactic vaccines and therapeutic drugs for this pandemic that is unprecedented in modern human history.
